# The Molecular Mechanisms of Action of Photobiomodulation Against Neurodegenerative Diseases: A Systematic Review

**DOI:** 10.1007/s10571-020-01016-9

**Published:** 2020-12-10

**Authors:** Mayukha Bathini, Chandavalli Ramappa Raghushaker, Krishna Kishore Mahato

**Affiliations:** 1 Manipal School of Life Sciences, Manipal Academy of Higher Education, Manipal, Karnataka 576104 India; 2 Department of Biophysics, Manipal School of Life Sciences, Manipal Academy of Higher Education, Manipal, Karnataka 576104 India

**Keywords:** Neurodegenerative diseases, Photobiomodulation, Molecular pathway, Neuroprotection

## Abstract

Neurodegenerative diseases might be slow but relentless, as we continue to fail in treating or delaying their progression. Given the complexity in the pathogenesis of these diseases, a broad-acting approach like photobiomodulation can prove promising. Photobiomodulation (PBM) uses red and infrared light for therapeutic benefits, working by stimulating growth and proliferation. The implications of photobiomodulation have been studied in several neurodegenerative disease models. It has been shown to improve cell survival, decrease apoptosis, alleviate oxidative stress, suppress inflammation, and rescue mitochondrial function. In in vivo models, it has reportedly preserved motor and cognitive skills. Beyond mitochondrial stimulation, the molecular mechanisms by which photobiomodulation protects against neurodegeneration have not been very well studied. This review has systematically been undertaken to study the effects of photobiomodulation at a molecular level and identify the different biochemical pathways and molecular changes in the process. The data showed the involvement of pathways like extracellular signal-regulated kinase (ERK), mitogen-activated protein kinase (MAPK), and protein kinase B (Akt). In addition, the expression of several genes and proteins playing different roles in the disease mechanisms was found to be influenced by PBM, such as neurotrophic factors and secretases. Studying the literature indicated that PBM can be translated to a potential therapeutic tool, acting through a spectrum of mechanisms that work together to decelerate disease progression in the organism, which is difficult to achieve through pharmacological interventions.

## Introduction

Neurodegenerative diseases like Alzheimer’s disease, Parkinson’s disease, and Huntington’s disease are characterized by the progressive structural or functional loss and death of neurons, eventually leading to a decline in cognitive, memory, and motor abilities. The pathogenesis of these diseases involves multiple overlapping aspects that are common among many neurodegenerative diseases, one of them, including protein aggregates. In Alzheimer’s, secretases generate amyloidogenic peptides (Aβ40 and Aβ42 are the most common isoforms) from the amyloid precursor protein (APP), which can misfold, aggregate to form extracellular amyloid plaques (Sanabria-Castro et al. 2017; Chen and Mobley 2019). And Parkinson’s disease, known to occur as a loss of dopaminergic neurons in the substantia nigra pars compacta, is attributed to lewy body aggregation containing the amyloid protein α-synuclein (α-syn) (Maiti et al. 2017). Polyglutamine (polyQ) diseases like Huntington’s disease and spinocerebellar ataxia are caused by a trinucleotide repeat expansion in the genes or polyglutamine tract expansion in the proteins, leading to misfolding and altered functions (Estrada Sánchez et al. 2008). The protein aggregates can interact with several mitochondrial components like cytochrome c oxidase (CCO), membrane translocases, voltage-dependent anion channels, and F_1_α, a subunit of ATP synthase (Hernandez-Zimbron et al. 2012; Pinho et al. 2014; Pozo Devoto and Falzone 2017). Reduced CCO activity, decreased mitochondrial membrane potential (MMP), and lower ATP generation are seen in brains of neurodegenerative disease patients. Mitochondrial complex I inhibitor 1-methyl-4-phenyl-1,2,3,6-tetrahydropyridine (MPTP) or 1-methyl-4-phenylpyridinium (MPP^+^) and the dopamine analogue 6-hydroxydopamine (6-OHDA) induce Parkinson’s disease-like lesions and symptoms in animal models (Chinta et al. 2010; Federico et al. 2012). Impaired cytochrome c oxidase function, usually accompanied by the antioxidant deficit, leads to oxidative stress. Reactive oxygen species (ROS) are further generated when the protein aggregates cause microglial activation, creating a pro-inflammatory environment. The presence of chronic inflammation also ends up being cytotoxic towards the neurons (Pagani and Eckert 2011; Chen and Zhong 2014). At the same time, the pathogenesis of Parkinson’s and Huntington’s is also due to loss of inhibition on cortical glutamatergic neurons and excess amounts of glutamate in the synaptic cleft due to interference of glutamate uptake by astrocytes, causing prolonged stimulation of glutamate receptors, a phenomenon called excitotoxicity. Excitotoxicity further worsens the mitochondrial dysfunction in neurodegenerative diseases, causing an intramitochondrial Ca^2+^ overload and triggers apoptosis by the release of cytochrome c through the mitochondrial transition pore. Mitochondrial dysfunction hence appears to be a central mediator of neurodegenerative disease pathogenesis and disease progression (Dong et al. 2009; Cerella et al. 2010; Vaarmann et al. 2013).

Photobiomodulation (PBM), also known as low-level laser therapy, is the application of light at red to infrared wavelengths for therapeutic purposes. The fluence delivered is low enough to prevent any thermal or ablative effects. The PBM relies on a photochemical mechanism. A wavelength of 650–1200 nm light is most commonly used for the application, absorbed by chromophores present in the cells. The cytochrome c oxidase (CCO), mitochondrial electron chain transport complex IV absorbs wavelengths from 600 to about 900 nm. Longer wavelengths are thought to be absorbed by water and light-sensitive ion channels such as the transient receptor potential (TRP) family, stimulating them to open. PBM can also reduce oxidative stress by dissociating the inhibitory nitric oxide (NO) from CCO (Hamblin 2016).

Due to its direct action on the mitochondria, PBM could be used as a potential therapeutic strategy for neurodegenerative diseases, which remain untreatable to date owing to the complex and layered aspects of the disease. PBM has been tested on neurodegenerative disease models such as Alzheimer’s disease, Parkinson’s disease, diabetic retinopathy, etc. In most cases, PBM shows beneficial effects by increasing mitochondrial function, reducing protein accumulation, reducing oxidative stress, and suppressing inflammation (Johnstone et al. 2016; Salehpour et al. 2018). PBM has mostly been used in Alzheimer’s and Parkinson’s disease animal models. PBM-treated animals showed improved behavioral and cognitive function, improved memory, and spatial learning compared to the deficits seen in neurodegenerative disease conditions (Oron and Oron 2016; Lu et al. 2017). Reduction in hyperphosphorylated tau, neurofibrillary tangles, and amyloid-beta (Aβ) plaques was also observed with PBM treatment (Purushothuman et al. 2014). In Parkinson’s disease models, PBM significantly reduced motor impairment and the animals had better control of their motor skills. PBM also facilitated the preservation of dopaminergic fibers in the animals’ brains (Oueslati et al. 2015). Additionally, PBM was found to decrease cerebrovascular damage after MPTP insult in mice (San Miguel et al. 2019).

For neurodegenerative diseases, the most commonly used PBM application mode is transcranial, where the light is applied directly to the head. However, there is also evidence that remote-PBM, where the light is applied to a part of the body distant from the intended target organ, is effective (Gordon and Johnstone 2019). A study by Ganeshan and colleagues found that remote-PBM preconditioning protects mice from MPTP-induced stress. The study found that preconditioning with PBM before MPTP administration mitigated the loss of dopaminergic neurons. The same study reported an investigation of transcriptomic changes in the brain after PBM treatment in normal animals, to understand how PBM made the animals more resistant to the neurotoxin. Multiple pathways were found to be involved in PBM therapy’s protection against MPTP-induced stress. Genes involved in cell proliferation and migration, and genes belonging to JAK/STAT, C-X-C chemokine receptor 4 (CXCR4), and NRF2-mediated oxidative stress response, which regulates key antioxidant genes, were upregulated (Ganeshan et al. 2019).

There is limited knowledge of the molecular mechanisms behind these effects. This systematic review aims to identify the mechanisms by which PBM alters neurodegenerative disease aspects like oxidative stress, inflammation, cell death, and protein aggregation and to establish its potential as a therapeutic intervention capable of affecting the disease as a whole system.

## Methodology

### Search Strategy

Searches were performed in the electronic databases of PubMed and Scopus in March 2020 using different terms (Table 1). There was no date restriction, and sorting was done by “Best Match” criterion on PubMed.

**Table 1:** Search terms used during the study

PubMed	(Low level laser therapy) AND neurons; (low level laser therapy[MeSH Major Topic]) AND neurodegenerative disease[MeSH Major Topic]; (low level laser therapy) AND neurodegenerative disease[MeSH Major Topic]; (low level laser therapy) AND neurogenerative disease; (photobiomodulation) AND neurons; (photobiomodulation) AND neurodegenerative disease; (photobiomodulation) AND neurodegenerative disease[MeSH Major Topic]; (low level laser therapy) AND parkinson’s disease; (photobiomodulation) AND parkinson’s disease; (low level laser therapy) AND parkinson’s disease; (photobiomodulation) AND parkinson’s disease; (low level laser therapy) AND huntington’s disease; (photobiomodulation) AND huntington’s disease; (low-level laser therapy) AND multiple sclerosis; (photobiomodulation) AND multiple sclerosis
Scopus	“Photobiomodulation” AND neurodegenerative disease; “low level laser therapy” AND neurodegenerative disease; “photobiomodulation” AND neurons; “low level laser therapy” AND neurons; “photobiomodulation AND parkinson’s disease”; “low level laser therapy” AND parkinson’s disease; “photobiomodulation AND huntington’s disease”; “low level laser therapy” AND huntington’s disease; “photobiomodulation AND parkinson’s disease”; “low level laser therapy” AND parkinson’s disease

### Inclusion Criteria

Original research articles, both in vitro and in vivo studies that quantitatively or semi-quantitatively assessed the influence of PBM on any particular signaling pathway, growth factors and neurotrophic factors, genes and proteins involved in apoptosis, differential expression of genes and proteins involved in amyloidogenic protein degradation and production pathways, cytochrome c oxidase, ATP, genes involved in mitochondrial functions, and up- or downregulation of mitochondrial proteins in the cells, tissue, or animal with induced neurodegeneration, were included.

### Exclusion Criteria

Reviews, book chapters, clinical trials, case reports, editorials, perspectives, letters, commentaries, reports, protocols, conference proceedings, foreign language, and research articles that did not use a photobiomodulation therapy on a test system/organism with induced neurodegeneration were excluded in the first phase after the title and/or abstract-based screening. Obtained full texts were further screened to exclude studies that did not fulfill the inclusion criteria. Those that did not clearly describe the method of administration of PBM were also excluded.

### Outcome Measures

The primary outcome measures chosen were the influence of PBM on proteins involved in signaling pathways and cascades, transcription factors, growth factors and neurotrophic factors, genes and proteins involved in apoptosis, cytochrome c oxidase, ATP, genes and proteins involved in mitochondrial functions, and differential expression of genes and proteins in amyloidogenic protein production and degradation. The secondary outcome measures were other mitochondrial characteristics reported, oxidative stress, cytokine expression, and differential expression of other relevant genes and proteins.

### Study Selection

Two authors independently performed title and abstract review, and full-text screening was carried out for studies remaining after phase 1 exclusion to identify the studies satisfying the inclusion criteria. Any differences were resolved by discussion and consensus.

### Data Extraction

In the studies meeting inclusion criteria, data were retrieved using a standard form to record the type of device, the wavelength used and fluence delivered, the test system or model organism used, a substance used to induce neurodegeneration, method of administration, control intervention, and outcomes. Due to the diversity in methodologies used and the results measured, a meta-analysis was not possible.

## Results and Discussion

**Fig. 1:**
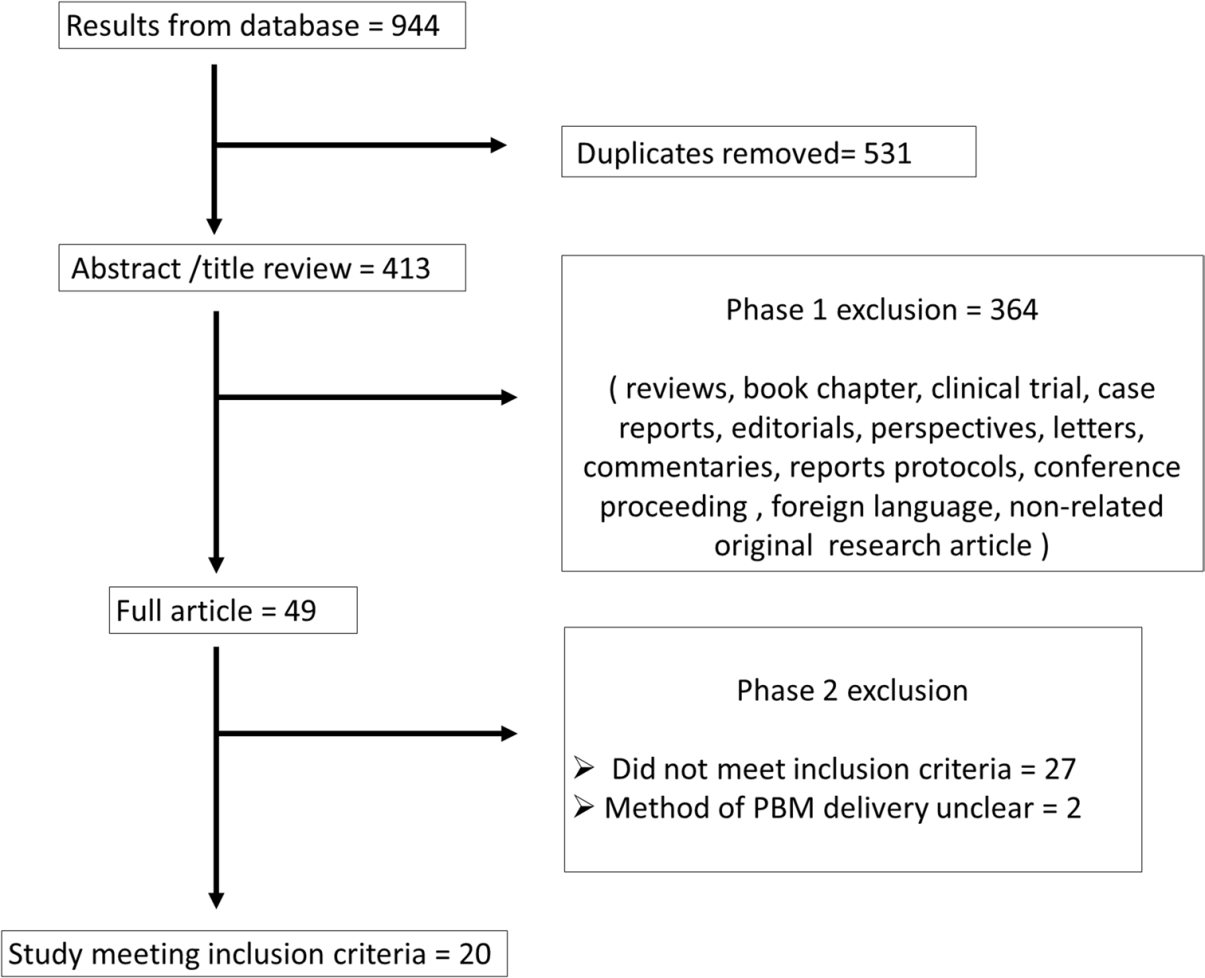
Flow diagram shows the selection process of eligible articles that applying photobiomodulation against neurodegenerative diseases

A total of 20 studies were finally included for data extraction and analysis in this review based on the inclusion criteria adopted. The workflow followed for the selection of eligible articles is shown in Fig. 1. Of these, 11 studies tested PBM with Alzheimer’s disease models, 2 used PBM with Parkinson’s disease models, 1 used PBM for in experimental autoimmune encephalomyelitis (a model of MS), and 2 assessed PBM for diabetic retinopathy. The four others did not use a model of any particular disease but used agents like hydrogen peroxide (oxidative stress), potassium cyanide and tetrodotoxin (neurotoxins), and NMDA, kainate, and glutamate (excitotoxicity) to induce neuronal cell degeneration (Table 2).

**Table 2:** The studies highlight the molecular pathways modulation induced by the photobiomodulation during neurological abnormalities

Serial number	Device and energy	Wavelength (nm)	Test System	Results	Reference
1	Helium -Neon (He-Ne) laser, 2 Joule (J)/cm^2^	632.8	Primary hippocampal neuronal cultures from APP/PS1 transgenic mice; hippocampal neurons from C57BL/6 mice; Aβ_1–42_, Aβ_25–35_ treated SH-SY5Y cell line	Ca^2+^/Ras/ERK/CREB pathway; Rac1 protein activated; Upregulation of BDNF, Postsynaptic density protein 95 (PSD-95)	Meng et al. (2013)
2	He-Ne laser, 2 J/cm^2^	632.8	MPP^+^ treated SH-SY5Y cell line	ERK/CREB pathway activated; Upregulation of vesicular monoamine transporter 2 (VMAT2); Dopamine content increased	Gu et al. (2017)
3	Light emitting diode (LED), 3 J/cm^2^	660	Organotypic hippocampal slice cultures from C57BL/6 mice; hippocampal cell line (HT-22) treated with Hydrogen peroxide (H_2_O_2_)	ERK/CREB pathway activated; Upregulation of BDNF; Increased activity of glutathione peroxidase, superoxide dismutase 1	Heo et al. (2019)
4	Diode laser via implanted optical fiber, 0.16 milliwatt (mW) for rats and mice and 10 mW in mice	670	MPTP injected BALB/c mice; MPTP injected macaque monkeys; 6-OHDA injected Wistar rats	Increased Glial cell line-derived neurotrophic factor (GDNF) in the monkey models	El Massri et al. (2017)
5	He-Ne laser, 2 J/cm^2^	632.8	Pheochromocytoma (PC-12) cells treated with Aβ_25–35_	Promoted β-catenin activation; Activated Akt inhibition of GSK3β	Liang et al. (2012)
6	He-Ne laser, 20 J/cm^2^	632.8	Lipopolysaccharide (LPS) activated microglia co-cultured with SH-SY5Y	PI3K/Protein kinase B (Akt) pathway activated through Src/Syk; Rac1 activated; Tumor necrosis factor (TNFα), inducible nitric oxide synthase (iNOS) decreased	Song et al. (2012)
7	He-Ne laser, 2 J/cm^2^	632.8	PC-12 cells treated with Aβ_25–35_	Akt/Yes-associated protein (YAP)-p73 pathway altered; Bax downregulated	Zhang et al. (2012)
8	He-Ne laser, 0.156 J/cm^2^–1.248 J/cm^2^	632.8	PC-12 cells treated with Aβ_25–35_	PKC mediated decrease in Bax/Bcl-xl ratio	Zhang et al. (2008)
9	He-Ne laser, 16.2 J/cm^2^	632.8	APPswe/PSENdE9 (APP/PS1) transgenic mice; SH-SY5Y-APPswe cells; primary hippocampal neurons from APP/PS1 mice	NADPH oxidase subunits gp91^phox^ and p47^phox^ assembly, cPLA2 phosphorylation inhibited; ROS, Interleukin 1 beta (IL-1β) and iNOS decreased	Yang et al. (2010)
10	He-Ne laser, 2 J/cm^2^	632.8	Swedish mutation of APP (APPswe)/APP/PS1 transgenic mice; SH-SY5Y-APPswe cells; primary hippocampal neurons from APP/PS1 mice	Cyclic adenosine monophosphate (cAMP)/Protein kinase A (PKA)/Sirtuin 1 (SIRT1) activated; A disintegrin and metalloproteinase domain—10 (ADAM10) upregulated, Beta-site APP cleaving enzyme 1 (BACE1) downregulated; CCO activity, MMP, ATP increased	Zhang et al. (2020)
11	Aluminum Gallium Arsenide (GaAlAs) LED array, 5 J/cm^2^	670	Myelin oligodendrocyte glycoprotein (MOG_35–55_) peptide immunized C57BL/6 mice	Downregulation of iNOS; Upregulation of Bcl2	Muili et al. (2013)
12	GaAlAs LED array, 5 J/cm^2^	670, 728, 770, 830, or 880	Postnatal rat visual cortex primary neurons treated with potassium cyanide, tetrodotoxin	CCO activity, ATP increased	Wong-Riley et al. (2005)
13	LED array, 5 J/cm^2^	670	C57BL/6 J mice with streptozotocin (STZ)-induced diabetic retinopathy	Vitamin D receptor (VDR) expression unchanged; Cytochrome P450 Family 24 Subfamily A Member 1 (Cyp24a1) gene expression increased; C-kit^+^ cells increased	Cheng et al. (2018)
14	LED probe, 2 J/cm^2^	610	5XFAD mice	Insulin-degrading enzyme (IDE) and Neprilysin (NEP) increased	Cho et al. (2018)
15	LED source, 4 J/cm^2^	670	K3 transgenic mice; APP/PS1 transgenic mice	CCO expression partially recovered and oxidative stress reduced	Purushothuman et al. (2014)
16	LED array, 6 J/cm^2^ (for in vivo experiments) and 5 J/cm^2^ (for in vitro experiments)	670	Lewis rats with STZ-induced diabetic retinopathy; retinal ganglion (RGC5), photoreceptor (661 W), Retinal Muller (rMC-1) cell line treated with high glucose	Akt, Heat shock protein (HSP27) and p38 mitogen-activated protein kinases (p38 MAPK) dephosphorylated; Intracellular adhesion molecule 1 (ICAM-1) decreased; No effect on Nitric oxide (NO) and Superoxide generation decreased	Tang et al. (2013)
17	LED array, 5 mW/cm^2^ for 6-min sessions	1072	TASTPM mice (APP/PS1 double transgenics)	HSP60, HSP70, HSP105, HSP27 and p-HSP27 increased; APP/PS1 decreased	Grillo et al. (2013)
18	GaAlAs diode laser, 10 mW/cm^2^, 50 mW/cm^2^ or 100 mW/cm^2^	808 ± 10	APP transgenic mice	ATP and mitochondrial oxygen consumption increased; c-fos expression increased; IL-1β, TGFβ, TNFα levels decreased	De Taboada et al. (2011)
19	Diode laser, 3 J/cm^2^	810	Primary mouse cortical neurons treated with glutamate, N-methyl-D-aspartate (NMDA) and kainate	ATP, MMP increased; Ca^2+^ content, ROS, NO decreased	Huang et al. (2014)
20	Diode laser, 15 J/cm^2^ at the cortex level	808	Sprague-Dawley rats given Aβ_1–42_ infusion	Bax/Bcl2 ratio, caspase-3 and 9, activity, oxidative damage decreased; Dynamin related protein 1 (Drp1) serine 616 phosphorylation, Mitochondrial fission 1 protein (Fis1), Mitochondrial fission factor (Mff), Mitochondrial dynamics protein MID51 (Mief), OPA1 mitochondrial dynamin-like GTPase (OPA1), Mitofusin 1 (MFN1) expression suppressed; IL-1β, Interleukin (IL-6), TNFα decreased; Increased MMP, ATP and CCO activity	Lu et al. (2017)

The data suggest that PBM alleviates the symptoms of neurodegenerative disease and slows down disease progression. PBM can preserve the normal state of the cells when stress is induced or influence cellular functioning to counter the neurodegenerative conditions. PBM therapy is capable of coaxing stressed neurons into producing neurotrophic factors like brain-derived neurotrophic factor (BDNF) and glial cell line-derived neurotrophic factor (GDNF) (Meng et al. 2013; Gu et al. 2017; Heo et al. 2019) and rescuing neurons from apoptosis by altering the expression and activity of pro- and anti-apoptotic proteins like Bcl2, Bcl-xL, Bax, and caspases (Zhang et al. 2008, 2012; Muili et al. 2013; Lu et al. 2017). It is also effective in reducing the amyloid plaque formation either by stimulating microglial phagocytosis of the protein aggregates or by altering the expression of enzymes involved in the amyloid peptide production and degradation pathways (Song et al. 2012; Zhang et al. 2020). The review also reaffirms the effect of PBM on improving the deterioration often seen in mitochondrial functioning in neurodegenerative diseases (Wong-Riley et al. 2005; De Taboada et al. 2011; Purushothuman et al. 2014; Huang et al. 2014; Lu et al. 2017; Zhang et al. 2020). The effects of PBM can be explained sequentially to begin with the stimulation of mitochondrial function leading to increased ATP, cAMP production, and Ca^2+^ signaling, which then activate several intracellular signaling molecules and pathways to alter gene and protein expression and activity.

### PBM Stimulates Mitochondrial Activity

PBM exerts a photochemical effect through the stimulation of mitochondrial cytochrome c oxidase (CCO). In neurodegenerative diseases, neurons experience mitochondrial dysfunction, loss of mitochondrial membrane potential (MMP), and depletion of ATP. PBM reverses the damage to the mitochondria fully or partially by stimulating CCO function and oxidative phosphorylation to restore MMP and raise ATP production (Wong-Riley et al. 2005; De Taboada et al. 2011; Huang et al. 2014; Zhang et al. 2020). It has been hypothesized that activation of CCO by PBM works by releasing nitric oxide (NO) from CCO, generating reactive oxygen species (ROS), and triggering NO signaling pathways (Hamblin 2016). This may not hold when PBM is used on cells already under stress (Huang et al. 2013), since the data reviewed here show a decrease in oxidative stress and NO signaling (Yang et al. 2010; Song et al. 2012; Huang et al. 2014).

### Mitochondrial Stimulation Triggers Several Signaling Pathways

PBM is known to activate multiple pathways such as ERK/CREB (Meng et al. 2013; Gu et al. 2017; Heo et al. 2019), cAMP/PKA/SIRT1 (Zhang et al. 2020), PKC (Zhang et al. 2008), Src/Syk/PI3K/Akt (Song et al. 2012), and Akt/GSK3β/β-catenin (Liang et al. 2012), involved in various molecular networks in the cellular system.

**Fig. 2:**
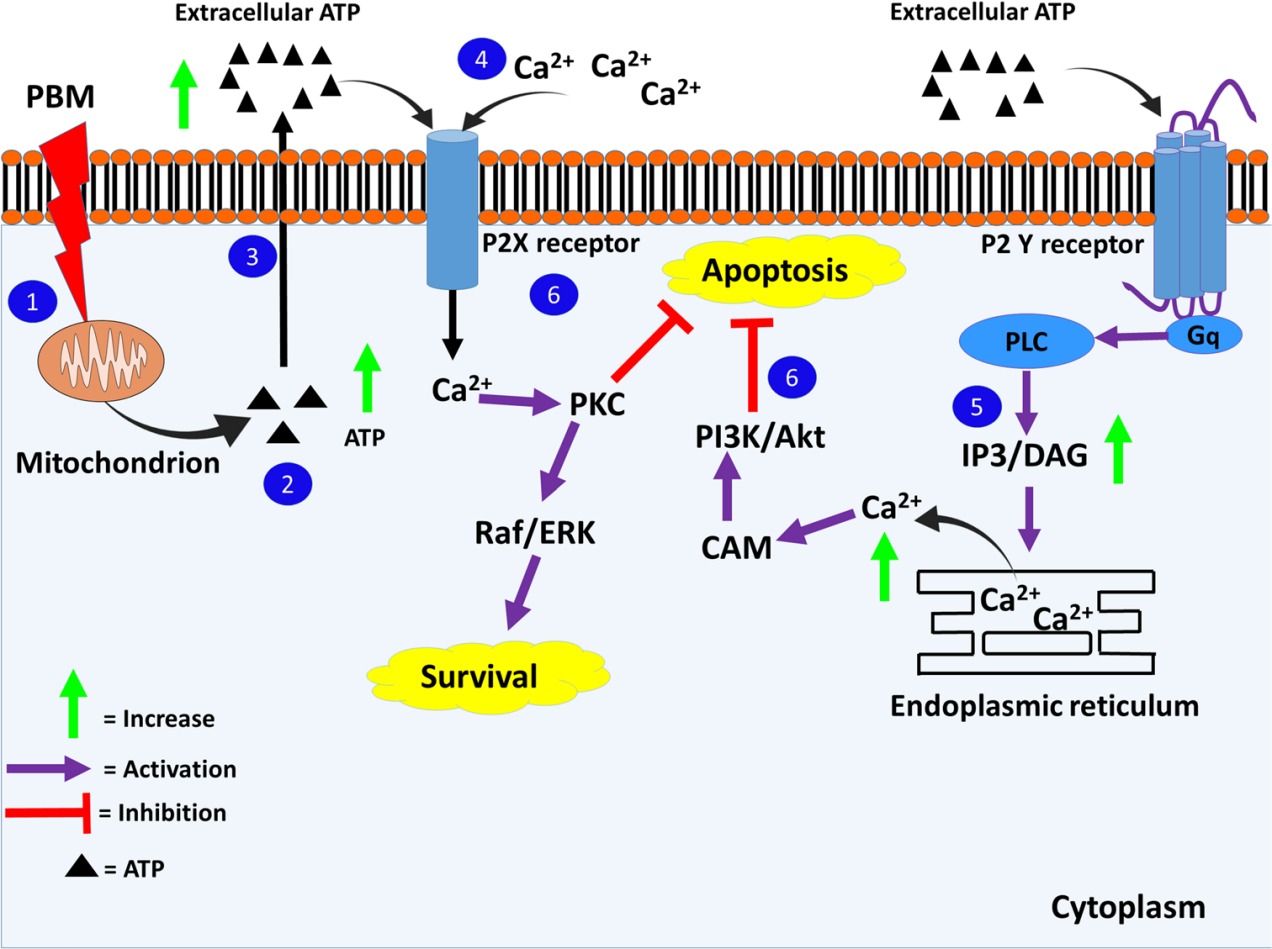
Effect of PBM through increased extracellular ATP and intracellular Ca^2+^. 1—Light is absorbed by mitochondrial CCO. 2—ATP production is increased. 3—ATP produced is secreted out and extracellular ATP increases. 4—Extracellular ATP binds to P2X receptor allowing Ca^2+^ influx. 5—Extracellular ATP binds to P2Y receptor which triggers the release of Ca^2+^ from endoplasmic reticulum (ER) stores. 6—Intracellular Ca^2+^ level increases, activating PKC and ERK pathways or PI3K/Akt through calmodulin (CaM)

**Fig. 3:**
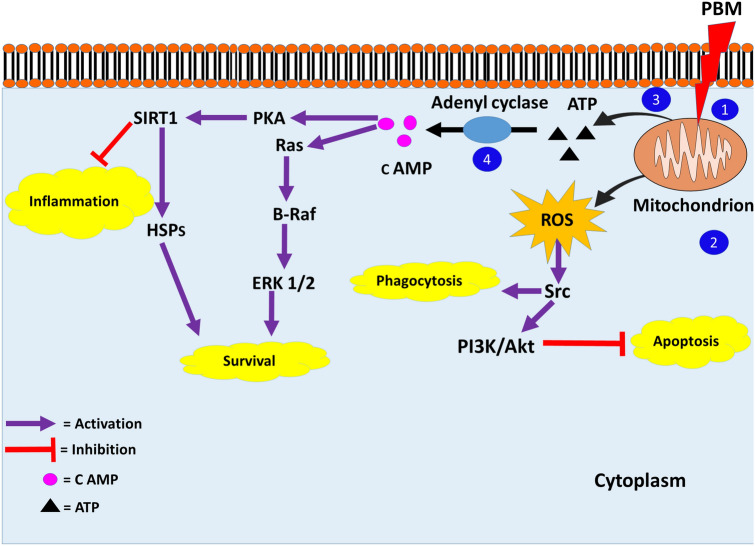
Effect of PBM through cAMP and ROS. 1—Light is absorbed by mitochondrial CCO. 2—ROS is produced and the ROS can activate Src and PI3K/Akt pathway. 3—ATP production is increased. 4—Adenyl cyclase converts ATP to cAMP. And the second messenger cAMP can activate PKA and Ras further leading to SIRT1 and ERK signaling

From the data, three significant paths through which the above-mentioned signaling cascades might be activated after PBM by CCO stimulation could be identified as follows:

1. Calcium ions (Ca^2+^)
  Increased ATP production and release due to CCO stimulation by PBM can induce Ca^2+^ influx through the purinergic P2X receptors. Extracellular ATP induces Ca^2+^ influx by binding to P2X receptors or triggering the production of IP_3_ through DAG when the ATP binds to P2Y receptors instead. Activated IP_3_Rs release Ca^2+^ from endoplasmic reticular (ER) stores (Wei et al. 2019). Ca^2+^ influx and increase in intracellular Ca^2+^ can activate PKC. PKC further leads to Raf/MEK/ERK signaling. Ca^2+^ can also activate PI3K/Akt through calmodulin (Moccia et al. 2019). In the data collected for this review, one study confirms that the activation of ERK pathway required Ca^2+^ release from intracellular stores (Fig. 2) (Meng et al. 2013). In contrast, PBM also acts as a neuroprotective against excitotoxic cell death by preserving the mitochondrial function and decreasing the toxic levels of intracellular Ca^2+^ content (Huang et al. 2014).
2. Cyclic adenosine monophosphate (cAMP)
  PBM stimulates the activity of CCO, raising MMP, and increasing the production of ATP in neurodegenerative disease models (Wong-Riley et al. 2005; Purushothuman et al. 2014; Huang et al. 2014). Adenyl cyclase can convert ATP to cAMP (Zhang et al. 2020). B-Raf is activated by cAMP through proteins like PKA, Ras, or Src. B-Raf can further activate ERK1/2. These mediators can also activate PI3K/Akt or SIRT1 (Dumaz and Marais 2005; Goldsmith and Dhanasekaran 2007). The above-mentioned study by Z. Zhang et al. confirmed the activation of SIRT1 through ATP and cAMP (Fig. 3) (Zhang et al. 2020).
3. Reactive oxygen species (ROS)
  CCO stimulation by PBM is hypothesized to be involved in NO release and generation of ROS. ROS signaling leads to the activation of pathways like NF-kB, NRF2, and PI3K/Akt (Fig. 3) (Zhang et al. 2016). Although none of the studies included for this review report an increase in NO signaling or ROS, an initial short-lived rise in oxidative species might be possible. PBM has been found to increase ROS when used in normal cell lines but decreases them in cells subjected to stress (Huang et al. 2013).

### Intracellular Signaling Molecules and Pathways

#### MAPK Pathway

A study by Chengbo Meng and others reported that intracellular Ca^2+^ levels rose by release from ER stores following PBM, which activated Ras kinase activity and thus the ERK/MAPK cascade in mouse Alzheimer’s disease model. The transcription factor CREB was the downstream effector of the Ras/ERK pathway (Meng et al. 2013). Activation of ERK/CREB after PBM was also confirmed by two other independent studies in HT-22 cells under oxidative stress and MPP^+^-treated SH-SY5Y cells (Gu et al. 2017; Heo et al. 2019).

One study investigated the phosphorylation of p38 in retinal ganglion (RGC5) cells line under high glucose conditions to test the use of PBM for diabetic retinopathy, where it was effective in suppressing the increased phosphorylation of p38 (Tang et al. 2013). P38 is a member of the MAPK family, inducible by pro-inflammatory cytokines and cellular stress like oxidative stress and radiation, and is a critical player in the regulation of apoptosis (De Zutter and Davis 2001; Yang et al. 2007). P38 MAPK is known to mediate apoptosis induced by the withdrawal of neurotrophic factors, often associated with neurodegenerative diseases (Kummer et al. 1997; Weissmiller and Wu 2012). Bim, a pro-apoptotic member of the Bcl2 family, is phosphorylated by p38 (Cai et al. 2006). P38 is also known to activate the pro-inflammatory NF-kB pathway (Hoesel and Schmid 2013). Hence, p38 dephosphorylation is beneficial in neurodegeneration.

#### PI3K/Akt Pathway

The PI3K/Akt pathway might be a focal point in PBM-stimulated effects. The pathway performs its anti-apoptotic functions at several levels, recruiting various downstream effectors. It inhibits p53-mediated apoptosis and enhances the transcription of anti-apoptotic genes by lifting the inhibition on nuclear factor kappa-light-chain-enhancer of activated B cells (NF-kB) and β-catenin, as well as inhibiting the forkhead box protein (FOXO) transcription factors that activate pro-apoptotic genes like Bcl2-like protein 11 (Bim) and BCL2-associated X (Bax) (Zhang et al. 2013). Akt increases anti-apoptotic Bcl2 expression by activating CREB/GATA-binding factor 1 (GATA1) (Jafari et al. 2019). Akt can also interfere with apoptosis by lifting the inhibition of hypoxia-inducible factor 1 (HIF-1) and increasing the transcription of heat shock protein (HSP) genes (Beurel and Jope 2006). An Akt pathway through which PBM prevents apoptosis is the Akt/GSK3β/β-catenin cascade. PBM activated Akt and its phosphorylation (inactivation) of GSK3β. The T cell factor (TCF)/lymphoid enhancer factor (LEF) proteins are the major end-point effectors of the Wnt/β-catenin signaling pathway, which promotes transcription of genes for survival (Liang et al. 2012; Herbst et al. 2014).

Another study reported PBM activation of PI3K/Akt as essential for protecting PC-12 cells from Aβ (25–35)-induced apoptosis (Zhang et al. 2012) through Yes-associated protein (YAP). In LPS-activated microglia, PBM brought about neuroprotective and anti-inflammatory responses, decreasing cytotoxicity to SH-SY5Y cells co-cultured with the LPS-activated microglia. The effects were reported to be mediated by the Src/Syk activation of the PI3K/Akt signaling pathway (Song et al. 2012).

#### SIRT1 Pathway

A member of the sirtuin family, SIRT1 is a deacetylase called into action during stress conditions, especially metabolic stress (Gerhart-Hines et al. 2011). PBM activated the cAMP/PKA/SIRT1 pathway in APP/PS1 transgenic mice, primary neurons, and SH-SY5Y cells containing the APPswe mutation. SIRT1 further deacetylated and activated the transcriptional inducer retinoic acid receptor beta (RAR-β) and the transcriptional coactivator PGC-1α (Zhang et al. 2020). There was a decrease in the production of Aβ peptides, while the expression of APP was unchanged. SIRT1 can work to suppress apoptosis by inhibiting the activity of p53, inducing HSPs through Heat shock factor 1 (HSF1), and activating the FOXO family of transcription factors (Raynes et al. 2013).

#### Heat Shock Proteins (HSPs)

Heat shock proteins are stress-induced proteins that mediate protein homeostasis and protein folding by acting as molecular chaperones (Meng et al. 2018). They suppress apoptosis caused by triggers such as oxidative stress, hyperthermia, cytotoxicity, and inflammation. Being essential for protein folding, the neuroprotective role of HSPs in neurodegeneration seems plausible given that many neurodegenerative diseases like Alzheimer’s disease, Parkinson’s disease, Huntington’s disease, amyotrophic lateral sclerosis, and spinocerebellar ataxia all have misfolded proteins as a common disease mechanism. HSP70, in particular, has been shown to decrease the aggregation of mutant proteins with expanded polyQ tracts like huntingtin and also of α-syn, Aβ, and superoxide dismutase 1 (SOD1) (Turturici et al. 2011). HSPs control apoptosis at several levels, such as regulating the expression of pro- and anti-apoptotic proteins, activating the PI3K/Akt pathway (Havasi et al. 2008) and NF-kB pathway (Meng et al. 2018) which further regulates anti-apoptotic genes such as Bcl2 (Hoesel and Schmid 2013), or inactivate the pro-apoptotic JNK pathway (Stetler et al. 2009). HSPs are also protectors against cell death caused by the withdrawal of neurotrophic factors, which are known to be required for the survival and growth of neurons. HSP105 has even demonstrated the ability to induce the production of the neurotrophic factor BDNF (Stetler et al. 2009; Hashikawa et al. 2017). PBM was shown in one study to have reduced amyloid peptide production and hyperphosphorylated tau in the Alzheimer’s disease model, TASTPM mice, by upregulating HSPs. HSP27 and p-HSP27, HSP60, HSP70, and HSP105 were found to be increased while no significant difference was observed in HSP40 and HSP90 (Grillo et al. 2013). Contrary to this, PBM in high glucose-treated RGC5 cells, a model for diabetic retinopathy, decreased p-HSP27, which was found to be increased in cells that did not receive PBM intervention (Tang et al. 2013). The opposite results seen in the two studies may be attributed to a difference in the disease, the model, as well as the PBM characteristics such as wavelength, 1072 nm, and 670 nm LED light, respectively.

Further, another signaling component found to be required for PBM’s anti-apoptotic effect is PKC. PBM was reported to protect against apoptosis in Aβ-treated PC-12 cells by activation of PKC (Zhang et al. 2008). It is known that PKC can modulate the activity of Bcl proteins by phosphorylating them directly and altering their interactions with Bax, or it may act by activating the Raf/MEK/ERK cascade (Deng et al. 2000).

Rat primary cortical astrocytes when pre-treated with PBM before exposure to Aβ_1–42_ experienced lesser oxidative stress and confirmed that PBM could decrease phosphorylated levels of the phospholipase cPLA2 and the activity of NADPH oxidase, a major contributor to oxidative stress. cPLA2 can lead to ROS production and inflammation through the synthesis of arachidonic acid, which further paves the way for the production of leukotrienes and prostaglandins. Hence, decreased p-cPLA2 is a mechanism of the anti-oxidative and anti-inflammatory effects of PBM (Yang et al. 2010; Sun et al. 2014).

### From Signaling Pathways to Therapeutic Effects

#### Maintenance and Activity of Neurons

PBM was found to attenuate dendrite atrophy caused by Amyloid-beta (Aβ) in hippocampal neurons. PBM raised the levels of brain-derived neurotrophic factor (BDNF), significantly reduced in the Alzheimer’s disease models, APP/PS1 transgenic mice, primary hippocampal neurons exposed to Aβ peptide, and hippocampal neurons from APP/PS1 transgenic mice embryos. BDNF was essential for the prevention of dendrite atrophy (Meng et al. 2013). BDNF is known to be involved in the growth and survival of neurons and neurogenesis and promotes excitatory synaptic transmission. Loss of BDNF negatively affects learning and memory (Binder and Scharfman 2004), and the upregulation seen with PBM may help not only the survival of the neurons but also alleviate cognitive symptoms of Alzheimer’s disease. The transcription of BDNF can be activated by Ca^2+^ influx and Ca^2+^-dependent activation of ERK and cAMP/PKA pathways (Zheng et al. 2012). The BDNF upregulation through Ras/ERK and binding of phosphorylated CREB to BDNF promoter was confirmed by the above-mentioned study in Alzheimer’s disease model and in another study of hippocampal neurons under oxidative stress (Meng et al. 2013; Heo et al. 2019). Glial cell line-derived neurotrophic factor (GDNF) was also found to be upregulated by PBM in monkey models of Parkinson’s disease (El Massri et al. 2017).

Vitamin D is also known to play a pivotal neuroprotective role against neurodegenerative disease pathogenesis. The vitamin D receptor (VDR) regulates the transcription of several genes essential for neuronal survival, functioning, and maintenance, such as the neurotrophic agents NGF and GDNF. VDR also protects against calcium excitotoxicity by regulating the expression of the L-type voltage-sensitive calcium channels. In addition, vitamin D also promotes Aβ phagocytosis (Gezen-AK et al. 2014; Banerjee et al. 2015). A study investigated the effect of PBM on VDR signaling in a model of diabetic retinopathy, the degeneration of retinal ganglion cells as a complication of diabetes. PBM raised the messenger RNA (mRNA) of CYP24A1, a gene regulated by VDR, compared to its subnormal expression in the disease condition. However, no difference was observed in the expression of VDR mRNA, suggestive of PBM influencing the VDR activity rather than expression. This area requires further investigation. The study also reported an increase in hematopoietic stem cells (CD45^−^/c-kit^+^) in circulation. Although it was not attributed to the beneficial effects of PBM against diabetic retinopathy, it confirms the mobilization of stem cells by PBM (Cheng et al. 2018).

PBM restored the dopamine content in MPP^+^ exposed cells by increasing the expression of the dopamine transporter VMAT2 and tyrosine hydroxylase, the enzyme responsible for producing dopamine. The expression of VMAT2, regulated by the ERK/CREB pathway, is essential for the maintenance and release of monoamines like dopamine and serotonin in monoaminergic neurons of the central nervous system and its expression, thus highlighting the neuroprotective role of PBM (Eiden and Weihe 2011; Gu et al. 2017; El Massri et al. 2017). Besides, PBM also improved neuronal transmission by upregulation of PSD-95, a scaffold protein required for assembly of neurotransmitter receptors and other signaling elements for postsynaptic transmission (Keith 2008; Meng et al. 2013). The expression of the transcription factor, c-fos, induced by neuronal activity (Chung 2015), was also raised by PBM (De Taboada et al. 2011).

##### Anti-inflammation

In LPS-activated microglia, PBM brought about neuroprotective and anti-inflammatory responses, decreasing cytotoxicity to SH-SY5Y cells co-cultured with the LPS-activated microglia. The effects were reported to be mediated by the Src/Syk activation of the PI3K/Akt signaling pathway. The effectors of the neuroprotective effect observed were myeloid differentiation primary response 88 (MyD88) and Ras-related C3 botulinum toxin substrate 1 (Rac1) proteins (Song et al. 2012). MyD88 is an adaptor protein involved in the signal transduction from toll-like receptors (TLRs) and IL-1 receptors (Deguine and Barton 2014). Rac1 is central to actin dynamics, essential for axonal growth and survival, and also plays a role in phagocytosis by promoting actin polymerization. Rac1 also has a function in regulating the ROS-producing nicotinamide adenine dinucleotide phosphate (NAPDH) oxidase (NOX) activity (D’Ambrosi et al. 2014). PBM promoted the degradation of MyD88, alleviating the pro-inflammatory conditions and activated Rac1, promoting F-actin accumulation and polymerization, in support of the phagocytic ability of the microglia (Song et al. 2012). The Rac1 activation might improve microglial phagocytosis of amyloid plaques.

The pro-inflammatory cytokines IL-1β, IL-6, TGFβ, and TNFα were found to be decreased by PBM (Yang et al. 2010; De Taboada et al. 2011; Song et al. 2012; Lu et al. 2017). These cytokines may be suppressed by p38 dephosphorylation seen with PBM (Bachstetter and Van Eldik 2010; Tang et al. 2013). In rat primary cortical astrocytes, PBM inhibited Aβ induced ROS from NOX (Yang et al. 2010). ROS from NOX can induce several molecules and signaling pathways like p38 MAPK/Phospholipase A2 (cPLA2), leading to inflammation (Sun et al. 2014). The study on astrocytes confirmed that PBM could decrease p-cPLA2. The ROS-producing nitric oxide synthase (iNOS) expression was also lowered (Yang et al. 2010; Song et al. 2012).

##### Anti-apoptosis

PBM prevented apoptosis by increasing Bcl2 and Bcl-xL, decreasing Bax and suppressing the activity of caspase-3 and caspase-9 (Zhang et al. 2008, 2012; Muili et al. 2013; Lu et al. 2017). In Aβ-treated PC-12 cells, the downregulation of Bax required PKC activation by PBM (Zhang et al. 2008). PBM also downregulated Bax through Akt activation and prevention of YAP-p73 complex formation (Zhang et al. 2012). YAP mediates apoptosis through its nuclear translocation and interaction with the transcription factor p73 and increases the expression of pro-apoptotic genes like Bax (Bertini et al. 2009). PBM activated Akt, which phosphorylates YAP and prevents YAP-p73 complex formation, suppressing the expression of Bax (Zhang et al. 2012).

Another identified mechanism through which PBM prevents apoptosis in Aβ-treated PC-12 cells is by lifting β-catenin inhibition. PBM, through the phosphorylation of Akt, leads to inactivation of GSK3β which normally inhibits β-catenin. PBM promoted β-catenin translocation to the nucleus and increased its transcriptional activity (Liang et al. 2012). Binding of β-catenin to the DNA-binding T cell factor/lymphocyte enhancer factor (TCF/LEF), causes the recruitment of coactivators and dismissal of corepressors normally bound to TCF/LEF in the absence of β-catenin and turns on the transcription of genes responsible for proliferation and survival such as MYC proto-oncogene (MYC) and ABCB1 (Herbst et al. 2014).

##### Antioxidant Capacity and Mitochondrial Preservation

Total antioxidant capacity was increased and the decrease in the antioxidants glutathione peroxidase (GPx), heme oxygenase 1 (HO-1), and superoxide dismutase (SOD1 and SOD2) was recovered following PBM treatment in animal models of Alzheimer’s disease and diabetic retinopathy, as well as SH-SY5Y cells under oxidative stress (Tang et al. 2013; Lu et al. 2017; Heo et al. 2019). PBM suppresses the pro-oxidant NO signaling by downregulating inducible NO synthase (NOS) (Yang et al. 2010; Song et al. 2012; Muili et al. 2013; Huang et al. 2014).

PBM also exerted its anti-oxidative effect by lowering the activity of glucose-6-phosphate dehydrogenase (G6PDH) that produces NADPH. Decreased availability of NADPH could be responsible for the lower activity of the ROS-producing NADPH oxidase (NOX) (Lu et al. 2017). While Aβ induced the assembly of NOX subunits gp91^phox^ and p47^phox^ and the production of ROS, PBM prevented their colocalization and attenuated ROS production (Yang et al. 2010). Though the study did not investigate the mechanism by which PBM prevented NOX assembly, it is known previously that the assembly of NOX subunits begins with phosphorylation of p47^phox^ by PKC (Rastogi et al. 2017).

The shift in mitochondrial dynamics towards the fission and fragmentation and functional loss of mitochondria is often seen in neurodegenerative diseases (Gao et al. 2017). In addition to improving mitochondrial oxidative phosphorylation by enhancing the activity of CCO, raising MMP, and improving ATP production, PBM affects the mitochondrial dynamics by altering the fusion and fission processes. PBM treatment preserves the expression of fusion proteins and prevents the shift towards fission and fragmentation. The expression of proteins controlling mitochondrial dynamics was influenced by PBM, where fission proteins like Fis1, Mff, and Mief were decreased. Drp1-S616 (serine 616) phosphorylation (fission promoting) was repressed, and Drp1-S637 (serine 637) phosphorylation (fusion promoting) was supported, Drp1-Fis1/Mff interactions were prevented, and expression and localization of the other fission proteins OPA1 and MFN1 were attenuated by irradiation, allowing the preservation of mitochondrial integrity (Wong-Riley et al. 2005; De Taboada et al. 2011; Purushothuman et al. 2014; Huang et al. 2014; Lu et al. 2017; Zhang et al. 2020). SIRT1, activated by PBM (Zhang et al. 2020), is also previously known to support mitochondrial biogenesis (Xu et al. 2018; Zhou et al. 2018). Hence, improvement in mitochondrial health through PBM therapy could also be through this mechanism.

##### Reduction in Amyloid Plaque Burden

PBM is effective in reducing the amyloid plaque burden either by stimulating microglial phagocytosis of the protein aggregates or by altering the expression of enzymes involved in the amyloid peptide production and degradation pathways (Song et al. 2012; Zhang et al. 2020). PBM stimulates microglia through Rac1 activation and promotes phagocytosis while promoting an anti-inflammatory environment, thus avoiding inflammation-induced cytotoxicity (Song et al. 2012). In addition, PBM stimulates GSK3β inhibition (Liang et al. 2012), which can further help towards preventing tau hyperphosphorylation (Zheng et al. 2002).

In APP/PS1 transgenic mice primary neurons and SH-SY5Y cells containing the APPswe, there was a decrease in the production of Aβ peptides, while the expression of APP was unchanged. Upon further investigation, the study reported that PBM activated the cAMP/PKA/SIRT1 pathway and altered APP processing by upregulating the α-secretase ADAM10 and downregulating the β-secretase BACE1. SIRT1 was responsible for these changes by deacetylating and activating retinoic acid receptor beta (RAR-β) and PGC-1α (Zhang et al. 2020). Retinoic acid receptor (RAR) alpha and beta are inducers of ADAM10 promoter activity (Endres and Deller 2017), while PGC-1α suppresses BACE1 expression (Katsouri et al. 2011; Wang et al. 2013). Also, PBM upregulated the Aβ degrading IDE and NEP enzymes in Alzheimer’s disease transgenic mice (Cho et al. 2018).

##### PBM Treatment Activates a Multidimensional Stress Response System in the Cells

The stress response action of PBM can be attributed to mainly a suppression of inflammation through dephosphorylation of p38 MAPK (Tang et al. 2013), inhibition of apoptosis through activation of SIRT1 (Zhang et al. 2020) and HSPs (Grillo et al. 2013), rescue from oxidative stress, and significant preservation of mitochondrial functioning (Huang et al. 2014; Purushothuman et al. 2014; Lu et al. 2017) as per previous studies results mentioned in this review. While the mechanism behind PBM regulation of antioxidant proteins is not well understood, we can postulate that the PKC and PI3K/Akt pathways modulated by PBM, also to some extent, control the anti-oxidant response by activation of NRF2/Anti-oxidant response element (ARE) signaling (Baird and Dinkova-Kostova 2011). PBM hence can support multiple cascades to preserve neuronal function and integrity and decelerate disease progression.

An interesting effect of PBM that remains unanswered is the mechanism by which PBM alters the expression and activity of the genes and proteins involved in mitochondrial dynamics to alleviate mitochondrial stress. It is well known that PBM decreases mitochondrial fragmentation and so it remains to be seen what factors play into the influence of PBM on mitochondrial dynamics (Lu et al. 2017; Wang et al. 2019).

A stress response mechanism commonly tested in PBM interventions is the NF-kB pathway. NF-kB is both pro-inflammatory and anti-apoptotic (Liu et al. 2017). Previous studies using PBM treatment for trauma and injury have shown that PBM decreases the activation of NF-kB (Rizzi et al. 2006; Lee et al. 2018). However, it is also postulated that ROS and NO generated by PBM treatment trigger the activation of NF-kB (Chen et al. 2011; Kumar et al. 2019). NO generation is hypothesized to occur when PBM activates CCO by displacement of NO, which generally inhibits CCO (Hamblin 2016). The included studies in the current review did not test the influence of PBM on NF-kB specifically. However, we can gather from the data that PBM suppressed NO signaling (Yang et al. 2010; Song et al. 2012; Muili et al. 2013; Huang et al. 2014), and these results may be extrapolated to say that NF-kB signaling might be suppressed under PBM influence in neurodegeneration conditions.

##### PBM Influences an Interconnected Network of Cellular Signaling Towards a Beneficial Effect

The data indicate that the effects of PBM are not mediated linearly by a single signaling cascade. PBM affects multiple pathways and multiple proteins, each of which often regulates very different functions in the same cell type. For instance, one of the proteins found to be activated by PBM in neurodegeneration models is the Rac1 GTPase (Song et al. 2012; Meng et al. 2013), a protein essential for axonal growth, stability, and phagocytosis. Rac1 inhibition causes actin dysfunction and motor neuron degeneration, but over-activation can cause the production of NOX-mediated ROS (D’Ambrosi et al. 2014), triggering NF-kB (Chen et al. 2011; Kumar et al. 2019), causing the production and release of pro-inflammatory cytokines and oxidative stress (Liu et al. 2017). Hence, the activation of Rac1 can either alleviate or exacerbate neurodegenerative disease conditions. The data from this review show two studies where PBM activated Rac1 (Song et al. 2012; Meng et al. 2013), but neither of the studies reported any pro-inflammatory effects nor an increase in ROS generation. A study by Sheng Song and colleagues suggested that ROS generated by PBM could be the activators of Src cascade; however, this was not investigated. The same study reported a decrease in pro-inflammatory cytokines and improved microglial phagocytosis (Song et al. 2012).

Similarly, PKC activation was reported to be responsible for the downregulation of Bax and upregulation of Bcl-Xl in PC-12 cells treated with Aβ_25–35_ (Zhang et al. 2008). From other available research studies, PKC is also an inducer of NOX; it activates the assembly of NOX subunits (Rastogi et al. 2017). The data from this review did not find any activation of NOX by PBM. In fact, PBM decreased the activity of NOX by preventing subunit assembly (Yang et al. 2010).

Available literature reports that ERK1/2 MAPK, PI3K/Akt, and SIRT1 cascades activate mitochondrial fission proteins like Drp1 and promote fission and fragmentation (Guedes-Dias and Oliveira 2013; Cook et al. 2017; Nagdas and Kashatus 2017). Though PBM activates the above pathways, data from this review demonstrate the preservation of mitochondrial fusion and a decrease in fission (Lu et al. 2017). It appears from these instances that when PBM activates a certain protein or pathway, it brings about beneficial changes without activating any other harmful effects of the pathways.

PBM also showed pathology-dependent effects. A study reported that PBM decreased p-Akt and p-HSP27 in a diabetic retinopathy model (Tang et al. 2013) while other studies reported an increase in p-Akt, HSP27, and p-HSP27 in activated microglia and Alzheimer’s disease models (Zhang et al. 2012; Liang et al. 2012; Grillo et al. 2013). While activation of Akt has been beneficial in Alzheimer’s disease (Liang et al. 2012; Zhang et al. 2012; Chen et al. 2016), it is quite the opposite in diabetic retinopathy where PI3K/Akt/mTOR activation does more harm than good (Jacot and Sherris 2011). This observation suggests that PBM’s effects would not be the same in different disease conditions.

Singularly targeting a particular signaling cascade is bound to fail, as seen with most pharmacological drugs for neurodegenerative diseases so far, since each pathway intersects with others to bring about a collective outcome. Suppressing or enhancing any one part of the complicated program can very often cause a whole different outcome. The p38, PI3K/Akt/GSK3β, and SIRT1 pathways are already under scrutiny as potential targets for pharmacological interventions against neurodegeneration. The efficacy of such compounds in humans is yet to be established. GSK3β inhibitor did not show much promise in the phase 2 trials while resveratrol, a SIRT1 activator shows some beneficial results (Munoz and Ammit 2010; Donmez and Outeiro 2013; Zhang et al. 2016; Sawda et al. 2017). Extensive clinical trials are required to judge whether they can succeed. Other than symptomatic treatments, most drugs aimed at slowing disease progression fail in human studies even though they showed promise in animal models (Kiaei 2013). The above-mentioned Rac1 and PGC-1α could be targets for pharmacological intervention, given the role of Rac1 in axonal survival (D’Ambrosi et al. 2014), and the importance of PGC-1α in mitochondrial biogenesis and suppression of Aβ peptide production (Katsouri et al. 2011; Wang et al. 2013; Zhang et al. 2020). PGC-1α is also found to be reduced in Alzheimer’s disease brains (Qin et al. 2009), and its overexpression as a treatment modality seems to offer some benefit but has also been reported to worsen Aβ and tau deposition and cause dopamine depletion (Clark et al. 2012; Dumont et al. 2014; Katsouri et al. 2016). Targeting the neurotrophic factors like BDNF and GDNF is also being tested for altering disease progression; however, their inability to cross the blood–brain barrier stands as a major ramification (Pilakka-Kanthikeel et al. 2013; Sathiya et al. 2013; d’Anglemont de Tassigny et al. 2015).

**Fig. 4:**
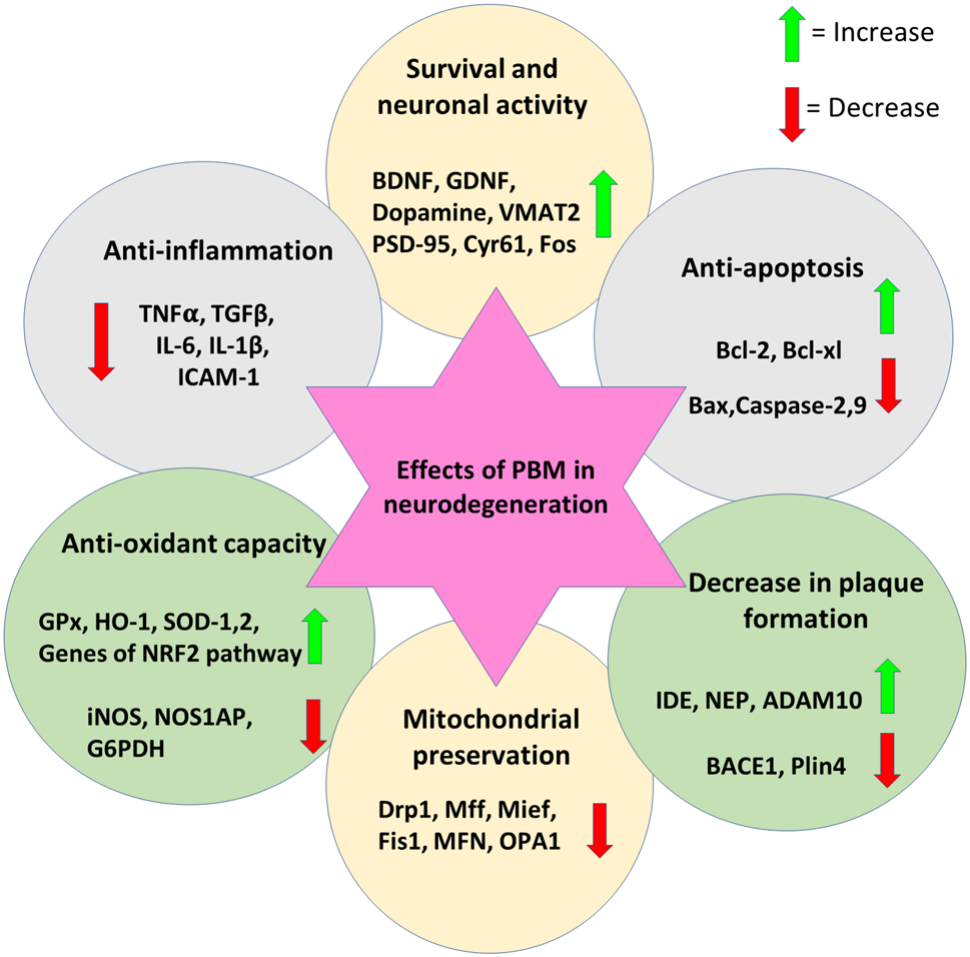
Overall effects of PBM on genes and proteins playing different roles in neurodegeneration

From the studies included in this review, it can be observed that PBM affects several intersecting pathways in a disease-dependent manner and nudges the molecular responses of cells in neurodegenerative diseases in the right direction (Fig. 4).

## Conclusion

In the present review, we have attempted to extensively highlight some of the significant molecular mechanisms by which photobiomodulation therapy is beneficial against neurodegenerative diseases. PBM modulates several pathways that are essential for stress response and neuronal survival. It improves neuronal survival and activity, attenuates inflammation, enhances the antioxidant capacity, and preserves mitochondrial homeostasis, thereby improving the neurons’ resilience and adaptability to cope with cellular insults during neurodegenerative disease progression. The review attempts to summarize the molecular aspects associated with PBM therapy, which could help researchers to better understand the neuronal conditions. The study report indicates that the application of photobiomodulation can be a promising tool for translation to therapeutic intervention, one that can alter the progression of neurodegenerative diseases.

## Abbreviations

ABCB1: ATP-binding cassette subfamily B member 1
p-Akt: (phosphorylated) protein kinase B
APPswe: Swedish mutation of APP
ATP: Adenosine triphosphate
Bcl2: B cell lymphoma 2
Bcl-xL: B cell lymphoma-extra large
CD45: Leukocyte common antigen
(p-)cPLA2: (phosphorylated) Cytoplasmic phospholipase 2
CREB: cAMP response element-binding protein
DAG: Diacylglycerol
GSK3β: Glycogen synthase kinase 3 beta
IP_3_: Inositol triphosphate
IP_3_R: Inositol triphosphate receptor
JAK: Janus kinase
JNK: c-Jun N-terminal kinase
mTOR: Mammalian target of rapamycin
NADPH: Nicotinamide adenine dinucleotide phosphate
NRF2: Nuclear factor erythroid 2-related factor
PGC-1α: Peroxisome proliferator-activated receptor-gamma coactivator 1 alpha
PI3K: Phosphoinositide 3-kinase
PKC: Protein kinase C
Raf: Rapidly accelerated fibrosarcoma protein
Ras: Rat sarcoma protein
STAT: Signal transducer and activator of transcription
Syk: Spleen tyrosine kinase
TASTPM: TAS10 x TPM double transgenic
TGF-β: Transforming growth factor beta
TNF-α: Tumor necrosis factor alpha
Wnt: Wingless-related integration site
